# Bactericidal Mechanisms of Chlorine Dioxide against Beta-Hemolytic *Streptococcus* CMCC 32210

**DOI:** 10.3390/cimb45060326

**Published:** 2023-06-13

**Authors:** Huan Liu, Jingju Zhang, Jing Liu, Guangjie Cao, Fei Xu, Xiubo Li

**Affiliations:** 1 National Feed Drug Reference Laboratories, Institute of Feed Research, Chinese Academy of Agricultural Sciences, Beijing 100081, China; 2 Key Laboratory of Animal Antimicrobial Resistance Surveillance, Ministry of Agriculture and Rural Affairs, Beijing 100081, China

**Keywords:** chlorine dioxide, disinfection, beta-hemolytic *Streptococcus*, mechanism

## Abstract

Chlorine dioxide is a globally recognized green and efficient disinfectant. This study aims to investigate the bactericidal mechanism of chlorine dioxide using beta-hemolytic *Streptococcus* (BHS) CMCC 32210 as a representative strain. BHS was exposed to chlorine dioxide, the minimum bactericidal concentration (MBC) values of chlorine dioxide against BHS were determined by the checkerboard method in preparation for subsequent tests. Cell morphology was observed using electron microscopy. Protein content leakage, adenosine triphosphatase (ATPase) activity, and lipid peroxidation were determined by kits, and DNA damage was determined using agar gel electrophoresis. The concentration of chlorine dioxide during disinfection showed a linear relationship with the concentration of BHS. Scanning electron microscopy (SEM) results showed that chlorine dioxide caused significant damage to the cell walls of BHS at a concentration of 50 mg/L, but had no significant effect on *Streptococcus* exposed to different exposure times. Furthermore, the extracellular protein concentration increased with increasing chlorine dioxide concentration, while the total protein content remained unchanged. The activities of Na^+^/K^+^-ATPase and Ca^2+^/Mg^2+^-ATPase decreased with increasing chlorine dioxide concentration. Chlorine dioxide treatment led to significant lipid peroxidation and DNA degradation in BHS. Leakage of intracellular components indicated that chlorine dioxide damaged the cell membrane of BHS. Chlorine dioxide exposure resulted in oxidative damage to lipids and proteins, which negatively impacted the cell wall and membrane of Streptococcus. This caused increased permeability and inactivation of key enzymes (Na^+^/K^+^-ATPase and Ca^2+^/Mg^2+^-ATPase) involved in respiratory metabolism, ultimately leading to DNA degradation and bacterial death due to either content leakage or metabolic failure.

## 1. Introduction

The use of disinfection products has attracted a great deal of attention in recent years [1]. The COVID-19 pandemic has tremendously increased the production and sales of disinfectants [2]. Chlorine dioxide is a strong oxidant with an antimicrobial activity that is often recommended for use as a disinfectant, even at low concentrations, to kill microorganisms [2,3,4]. As such, chlorine dioxide is a broad-spectrum [5], safe, and efficient disinfectant [6,7] due to its high oxidation efficiency and its ability to reduce the formation of organic chlorination by-products during the application, and has been used in a wide range of applications including disinfection of water [8] and wastewater [9], medical devices [10,11], agricultural products [12,13,14], and Stomatology [15].

Notably, chlorine dioxide gas is unstable and not easily stored [16], limiting its promotion in disinfectants. Our research team has created a stable chlorine dioxide solution with levels up to 500 mg/L, which is highly efficient and stable and easy to store [17]. Chlorine dioxide solution can be used to rapidly eliminate pathogenic microorganisms in public and breeding environments, providing an effective means for public health and the well-being of humans and animals. In a previous study, the disinfection efficacy of a stabilized chlorine dioxide product was assessed against *Staphylococcus aureus*, *Escherichia coli*, BHS, and *Bacillus subtilis* [18]. Additionally, the product’s ability to inactivate the Thiveral strain of swine fever virus was investigated [18], and the results demonstrated its excellent disinfection performance.

Research on the mechanism of bacterial virus killing by chlorine dioxide has attracted widespread attention. Earlier research indicated that chlorine dioxide can act as a bactericidal agent against several types of bacteria, including *Escherichia coli* [19], *Staphylococcus aureus* [20], and *Bacillus subtilis* [21]. Its mechanism involves reducing the permeability of both outer and inner membranes of bacterial cells, which results in the release of important nuclear materials associated with cell viability loss or death.

BHS is an important streptococci that forms a 2–4 mm wide hemolytic ring around its colonies due to the production of hemolysin toxin. [22,23], and can cause a variety of diseases in humans [24] and animals [25]. To our knowledge, studies have reported the antibacterial activity of chlorine dioxide against BHS [26,27], but the mechanism of killing BHS has been rarely reported. Thus, in our study, BHS CMCC 32210 was selected, and the MBC (minimum bactericidal concentration) values of chlorine dioxide against BHS were measured. The bactericidal mechanism of chlorine dioxide on BHS by measuring changes in cell morphology, protein leakage, ATPase activity, lipid oxidation, and DNA damage were identified. 

## 2. Materials and Methods

### 2.1. Bacterial Strains and Preparation of Suspension

BHS strain 32210 was obtained from the National Medical Culture Collection. Frozen samples were resuspended in 10 mL of sterile nutrient broth containing 3% fetal bovine serum (FBS), thawed and mixed, and incubated at 37 °C for 18–24 h. The first generation was extracted from the culture suspension using an inoculation loop; individual bacterial cells were isolated by striking out on Luria–Bertani (LB) agar plates containing 5% sterile defibrinated sheep blood. Next, the individual bacterial colonies obtained were inoculated into an LB broth medium containing 3% FBS for bacterial enrichment. During enrichment, the medium was incubated overnight (18–24 h) in a constant temperature oscillating incubator (37 °C, 200 rpm) [28].

### 2.2. Chlorine Dioxide Preparation and Measurement

Sodium chlorite was reacted with an acid activator in a generator to produce gas, which was then continuously collected into a liquid containing a stabilizer. When the chlorine dioxide concentration reached 500 mg/L, its absorption was stopped, and a stable chlorine dioxide solution was formed.

The content of the chlorine dioxide disinfectant was determined using the five-step iodometric method, and it was subsequently diluted to the required concentration using sterilized deionized water [29].

### 2.3. Determination of MBC Values

Plates were counted for plate-stage BHS after 10-fold dilution. A 12 × 12 checkerboard was designed on two 96-well plates with a twofold gradient dilution of 50 μL per well starting from the plateau concentration of *Streptococcus* CMCC 32210 to the last well [30]. Furthermore, 50 μL of chlorine dioxide solution was added to each well to give a final concentration of 0.154–315 mg/L of chlorine dioxide in 100 μL of reaction solution per well. A neutralizing agent (containing 0.3% Na_2_S_2_O_3_, 1.0% Tween-80, 0.1% lecithin in LB broth, and 5% FBS) was added for 10 min at room temperature (25 ± 2 °C). Neutralizer has no effect on the growth of the bacterium [29]. The LB broth (containing 0.3% Na_2_S_2_O_3_ 1.0% Tween-80, 0.1% lecithin, and 5% FBS) was shaken uniformly for 10 min and then incubated in a constant temperature incubator at 37 °C for 20–24 h. Standard hard water was used as the positive control instead of a disinfectant, and wells with phosphate-buffered saline (PBS) instead of bacterial solution were used as the negative control. Next, 100 μL of the bacterial solution from each well was applied to LB agar plates containing 5% FBS, and the concentration and MBC of the bacterial solution with a 100% killing rate were recorded.

### 2.4. Morphological Analysis and Cytoplasmic Membrane Integrity Assays

SEM was used to observe the ultrastructural changes in bacteria before and after treatment with the disinfectants (H7650, Hitachi Limited, Tokyo, Japan). The 2.5 mL bacterial solution was treated with 50 mg/L and 10 mg/L chlorine dioxide (the control group used PBS instead of chlorine dioxide), and the mixture was thoroughly combined with the neutralizing agent after 30 s, 5 min, and 10 min. Next, 0.1 mL of the mixture was taken for viable cell counting, the neutralization product was centrifuged at 6000 rpm for 10 min (D3024R, Scilogex, CA, USA), the supernatant was discarded, and the residue was cleaned three times with PBS. The samples were then fixed overnight with glutaraldehyde solution at 4 °C, centrifuged at 3000 rpm for 10 min, and the precipitation was detected using SEM [17]. 

### 2.5. Intracellular Protein Leakage Assays

Referring to the MBC curve, BHS at a concentration of 10^9^ CFU/mL were exposed to 10, 50, and 100 mg/L of chlorine dioxide. Neutralizing agents were added at 1, 5, 10, and 30 min, and sterile distilled water was used instead of chlorine dioxide solution for the control group. The treated samples were centrifuged at 6000 rpm for 5 min, the supernatant was discarded, and 1 mL of saline was added to the sample and stored at 4 °C [31]. 

The extracellular protein concentration and total protein after lysis of BHS exposed to different concentrations of chlorine dioxide were measured at 562 nm in strict accordance with the Bicinchoninic acid (BCA) protein concentration assay kit (P0009, Beyotime Biotechnology, Shanghai, China).

### 2.6. ATPase Activity Assays

Leakage of Na^+^/K^+^ ATPase and Ca^2+^/Mg^2+^ ATPase, which are representatives of intracellular components, was detected using the intracellular component leakage assay. BHS at a concentration of 10^9^ CFU/mL were exposed to 10, 50, and 100 mg/L of chlorine dioxide with reference to the MBC curve. Neutralizing agents were added at 1, 5, 10, and 30 min, and sterile distilled water was used instead of chlorine dioxide solution for the control group. BHS from the neutralized bacterial suspension prepared above was centrifuged, and the supernatant was discarded, and then the bacteria were crushed using an ultrasonic crusher (KQ-500DE, Kun Shan Ultrasonic Instruments, Jiangsu, China). Ultrasonic crushing method: 2 mL of the bacterial solution was centrifuged, suspended with 200 μL of buffer, 35 μL of lysozyme was added, the mixture was placed on an ice bath for 30 min and then sonicated for at least 30 min (750 W 40% power, 5 s sonication, and 9 s interval) [32]. The concentration of protein from BHS were measured by the BCA Protein Assay Kit, and the bacterial homogenate was then diluted to different concentrations for pretesting. The resulting absolute absorbance value of approximately 0.2 was used as the sampling concentration.

Assays were performed using ultra micro Na^+^/K^+^ ATPase and Ca^2+^/Mg^2+^ ATPase test kits (SW-CJ-2FD, Nanjing Jiancheng Institute of Biological Engineering, Nanjing, China). The samples were treated according to the kit procedure and left at room temperature (25 ± 2 °C) for 5 min, and the absorbance value of each tube was determined at 636 nm using a 1 cm optical diameter and double distilled water for zeroing (UV-2550, Shimadzu, Guangdong, China). 

### 2.7. Lipid Peroxidation Assays

Malondialdehydes, MDA, is an important factor to evaluate the lipid peroxidation. BHS at a concentration of 10^9^ CFU/mL were exposed to 10, 50, and 100 mg/L of chlorine dioxide with reference to the MBC curve. Neutralizing agents were added at 1, 5, 10, and 30 min, and sterile distilled water was used instead of chlorine dioxide solution for the control group. Next, 0.1 mL of the homogenate per 10 million bacteria was used as a homogenate for bacterial lysis, 2 mL of bacterial solution was centrifuged, suspended with 200 μL of saline, 35 μL of lysozyme was added, the mixture was placed on an ice bath for 30 min (ST-1568, Beyotime Biotechnology, Beijing, China) and then sonicated for at least 30 min (750 W 40% power, 5 s sonication, and 9 s interval). After lysis, the supernatant was removed using centrifugation at 10,000–12,000 rpm for 10 min for subsequent determination. The protein concentration was determined using the BCA protein concentration assay kit (for subsequent calculation of the MDA content per protein weight in tissues or cells), and the MDA was determined using the lipid peroxidation assay kit (S0130S, Beyotime, Beijing, China) for the treated BHS [33]. Samples were treated according to the kit procedure, the absorbance values of the sample, and the standard reaction solution were measured at 535 nm using an enzyme marker (Epoch2, BioTek, VT, USA), a standard curve was established. The content of MDA was calculated according to the standard curve formula.

### 2.8. Gel Electrophoresis and DNA Damage Analysis

BHS at a concentration of 10^9^ CFU/mL were exposed to 10, 50, and 100 mg/L of chlorine dioxide with reference to the MBC curve. Neutralizing agents were added at 1, 5, 10, and 30 min, and sterile distilled water was used instead of chlorine dioxide solution for the control group. BHS were extracted from the neutralized bacterial solution (DP302-02, Tiangen Biotech, Beijing, China) through centrifugation at 3000 rpm for 10 min at 4 °C. DNA was isolated using gel electrophoresis (JM-250, Jim-X Scientific Company, Dalian, China) on 0.8% agarose in TAE buffer, which was prepared by adding Tris (242 g) and EDTA (18.612 g) to a 1 L beaker, mixing well with approximately 800 mL of deionized water, adding glacial acetic acid (57.1 mL), and adjusting the pH with NaOH until it reached 8.0. Deionized water was then added to fix the volume to 1 L, and the mixture was stored at room temperature [34]. After staining with nucleic acid dye working solution (Beijing solarbio science and technology, Beijing, China), the DNA was visualized using a gel imaging system (Beijing YuanPingHao Biotech, Beijing, China).

### 2.9. Statistical Analysis

Statistical analysis was performed using SPSS software (version 26.2, SPSS Inc., Chicago, IL, USA) and visualized using GraphPad Prism 8. A significant difference was defined when *p* < 0.05.

## 3. Results

### 3.1. MBC Values

**Figure 1:**
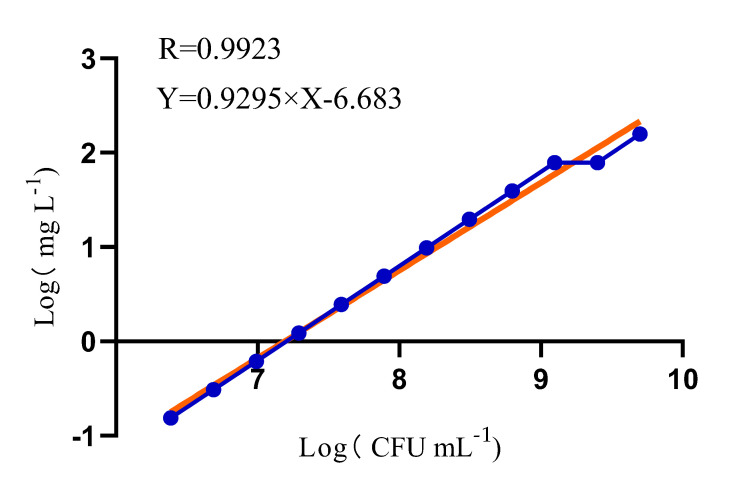
Chlorine dioxide killing curve against BHS.

The MBC values of chlorine dioxide were determined using the checkerboard twofold dilution method for 12 concentration gradients from 10^6^–10^9^ CFU/mL, and the results in Figure 1 show that the bactericidal effect of chlorine dioxide is concentration-dependent and consistent with the linear equation (R^2^ = 0.9923) (Figure 1). In a subsequent study of the bactericidal mechanisms, we chose a concentration of 10^9^ and used 50 mg/L chlorine dioxide as the medium concentration, and 10 mg/L and 100 mg/L as the low and high concentrations, respectively.

### 3.2. Ultrastructural Changes in BHS Treated with Chlorine Dioxide

**Figure 2:**
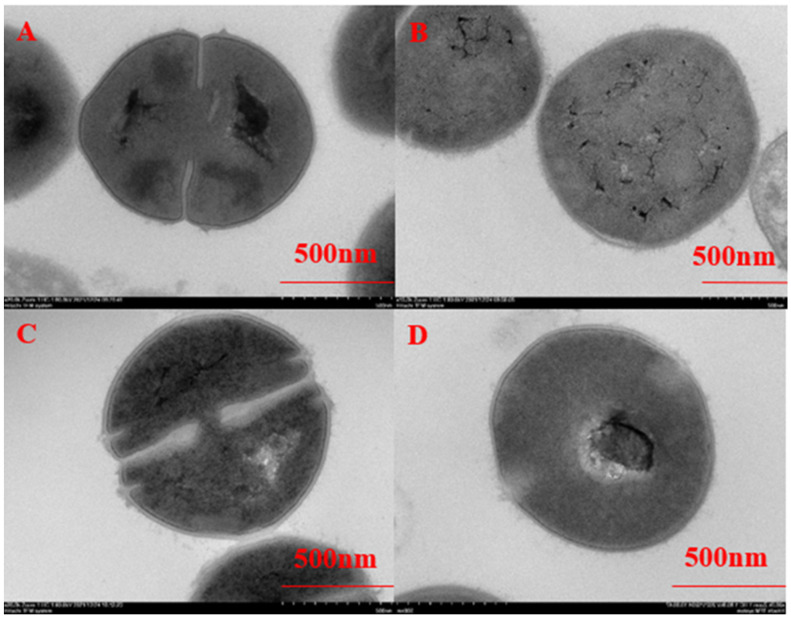
Ultrastructural changes in BHS before and after the 10 mg/L chlorine dioxide action ((**A**–**D**) represent treatment at 0, 0.5, 5, and 10 min, respectively).

**Figure 3:**
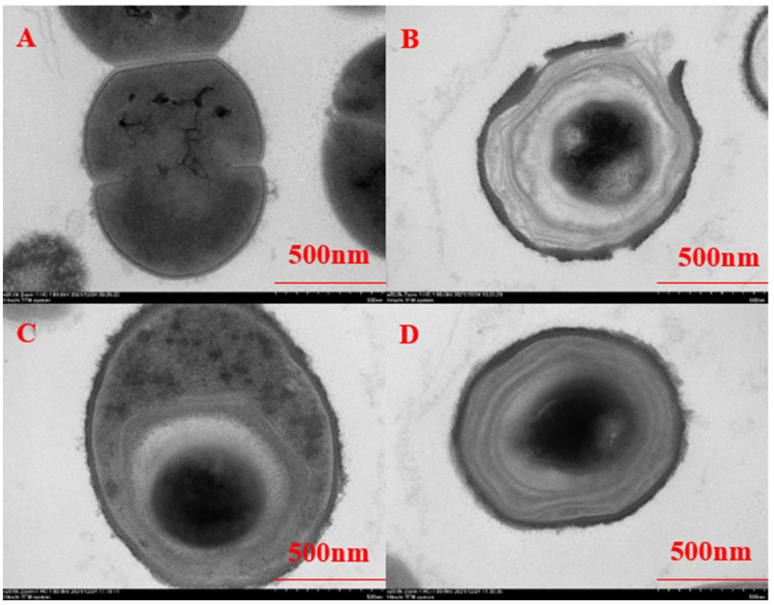
Ultrastructural changes in BHS before and after the 50 mg/L chlorine dioxide action ((**A**–**D**) represent treatment at 0, 0.5, 5, and 10 min, respectively).

The effect of chlorine dioxide solution on the cell morphology of BHS was assessed using SEM by comparing untreated bacteria and bacteria exposed to different concentrations of chlorine dioxide disinfectant at different times. In the control group, streptococcal cell walls were smooth and clear, with cell membranes fitting closely to the cell walls and evenly distributed cytoplasm (Figure 2A and Figure 3A). When exposed to a concentration of 10 mg/L chlorine dioxide, BHS showed slight separation of the mass wall and more evenly distributed contents, but did not significantly affect streptococci (Figure 2B–D). However, exposure to a concentration of 50 mg/L chlorine dioxide resulted in significant changes, including increased cell thickness, cell wall breakage, an increase in the outer villi-like material on the cell wall, deep crumpling of the cell contents, and cytoplasmic leakage (Figure 3B–D). There was no significant effect of different reaction times on BHS. Chlorine dioxide may promote the release of intracellular components and disrupt cellular homeostasis. 

### 3.3. Bacterial Intracellular Protein Leakage Assay

**Figure 4:**
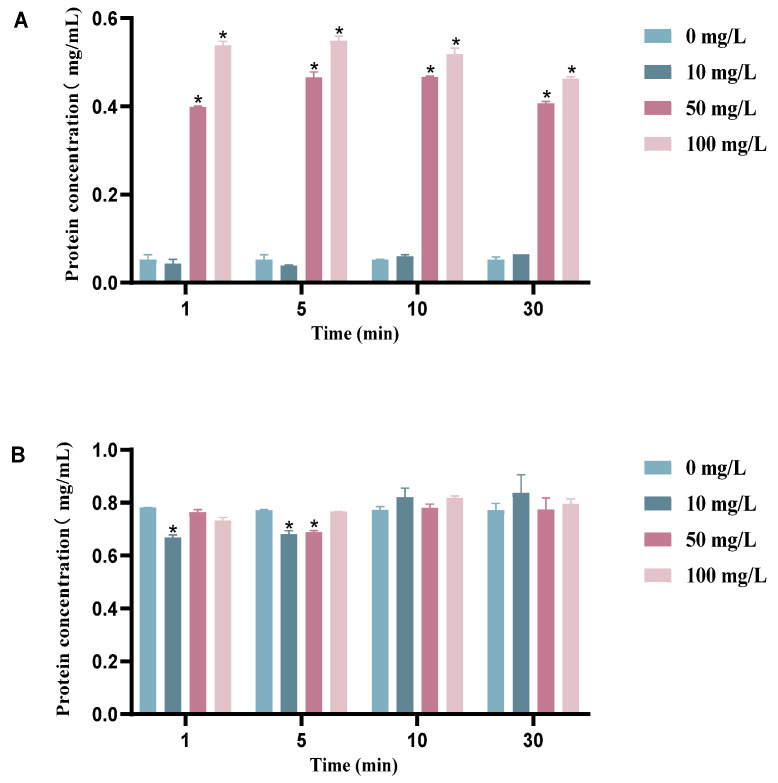
Protein concentration determination. (**A**) Extracellular BCA protein concentration of BHS after treatment with different concentrations of chlorine dioxide; (**B**) BCA protein concentration after lysis of BHS treated with different concentrations of chlorine dioxide. Significant difference between control group and chlorine dioxide treatment group was indicated by asterisks as * *p* < 0.05. The bars indicate the mean ± standard error of mean (n = 3).

The extracellular protein concentrations of BHS after different treatments are shown in Figure 4A, while the total protein concentrations inside and outside the bacterial cell after lysis are shown in Figure 4B. On Figure 4A, it can be seen that the extracellular protein concentration of BHS increased with increasing chlorine dioxide concentration; additionally, the differences in extracellular protein concentrations of BHS at 50 mg/L and 100 mg/L chlorine dioxide concentrations were not significant, compared to the 10 mg/L concentration and the blank groups.

The difference between the extracellular protein concentration of BHS and the blank group is not significant (*p* > 0.05) at 10 mg/L chlorine dioxide, indicating that the leakage of intracellular protein increased with an increase in chlorine dioxide concentration, but the difference in extracellular protein concentration of streptococci is not significant at the same concentration of chlorine dioxide for different times (Figure 4). The lysis treatment of each group of BHS shows little difference in the total protein concentration inside and outside the BHS after different concentrations of chlorine dioxide treatment, which also proves that the extracellular protein in the 50 mg/L and 100 mg/L concentration groups originated from intracellular leakage and increased cell membrane and cell wall permeability. We also found that the duration of chlorine dioxide exposure did not have a significant impact on the results of protein leakage.

### 3.4. Results of Intracellular ATPase Activity Assay of Bacteria

**Figure 5:**
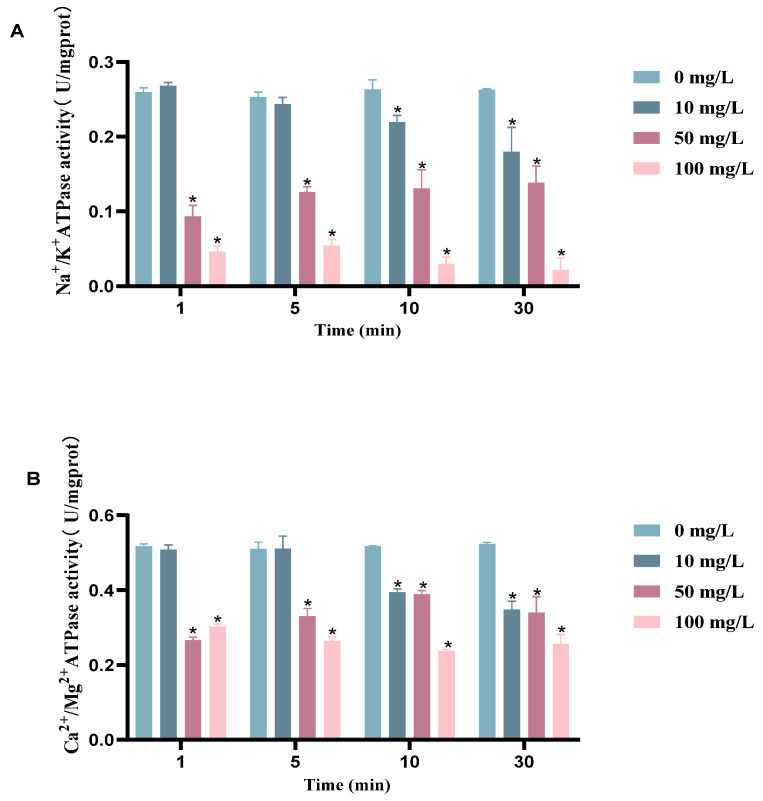
ATPase activity of BHS before and after chlorine dioxide treatment. (**A**) Na^+^/K^+^-ATPase activity of BHS before and after chlorine dioxide treatment; (**B**) Ca^2+^/Mg^2+^-ATPase activity of BHS before and after chlorine dioxide treatment. Significant difference between control group and chlorine dioxide treatment group was indicated by asterisks as * *p* < 0.05. The bars indicate the mean ± standard error of mean (n = 3).

**Figure 6:**
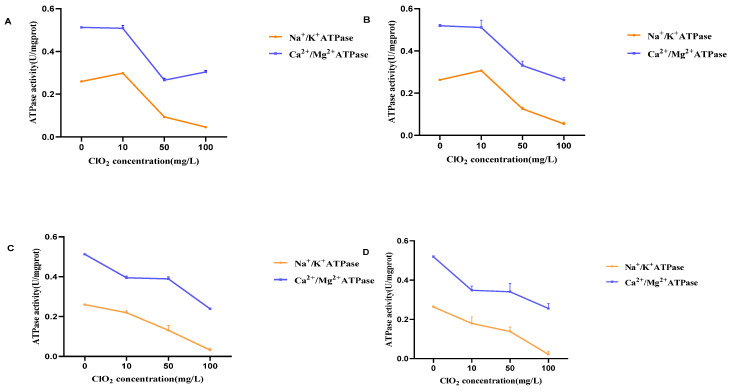
(**A**) Na^+^/K^+^-ATPase and Ca^2+^/Mg^2+^-ATPase activities of BHS at different concentrations of chlorine dioxide for 1 min. (**B**) Na^+^/K^+^-ATPase and Ca^2+^/Mg^2+^-ATPase activities of BHS at different concentrations of chlorine dioxide for 5 min. (**C**) Results of Na^+^/K^+^-ATPase and Ca^2+^/Mg^2+^-ATPase activities of BHS at different concentrations of chlorine dioxide for 10 min. (**D**) Na^+^/K^+^-ATPase and Ca^2+^/Mg^2+^-ATPase activities of BHS at different concentrations of chlorine dioxide for 30 min. The bars indicate the mean ± standard error of mean (n = 3).

Significant differences were found between the control and Na^+^/K^+^-ATPase at 50 mg/L and 100 mg/L chlorine dioxide concentrations. The Na^+^/K^+^-ATPase and Ca^2+^/Mg^2+^-ATPase were significantly different compared to the control when BHS was exposed to a concentration of 10 mg/L chlorine dioxide for an extended time of 10 min. The activities of BHS Na^+^/K^+^-ATPase and Ca^2+^/Mg^2+^-ATPase were decreased gradually with increasing concentrations of chlorine dioxide treatment (Figure 5A,B). The activities of both ATPases decreased with increasing chlorine dioxide concentrations (Figure 6). Therefore, the higher the concentration of chlorine dioxide, the more severe the ATPase inactivation. The duration of chlorine dioxide exposure did not show a clear pattern in its effect on ATPase activity.

### 3.5. Results of Lipid Peroxidation Assay in Bacterial Cells

**Figure 7:**
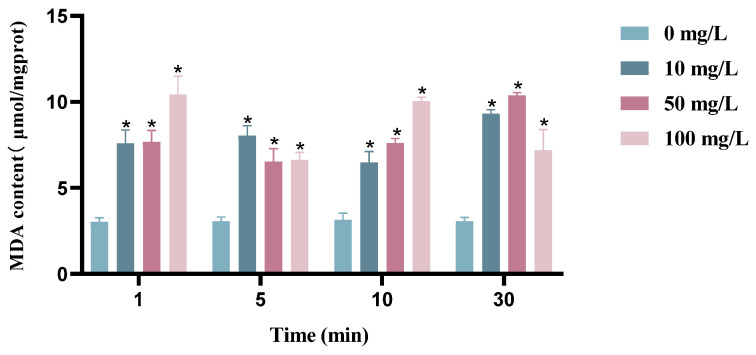
MDA content measurement of BHS before and after chlorine dioxide treatment. Significant difference between control group and chlorine dioxide treatment group was indicated by asterisks as * *p* < 0.05. The bars indicate the mean ± standard error of mean (n = 3).

Different concentrations of chlorine dioxide solutions acting on BHS show a significant increase in lipid peroxide product (MDA) compared with the blank group, which proves that chlorine dioxide has an oxidizing effect on BHS. However, the difference in MDA content was not significant for different time treatments of BHS (Figure 7).

### 3.6. Determination of DNA Damage in Streptococcal Cells

**Figure 8:**
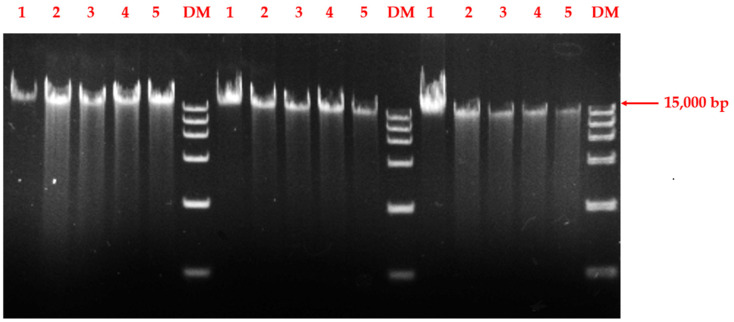
Images of DNA gel electrophoresis after different treatment times of chlorine dioxide. Lane 1 represents no contact with chlorine dioxide. Lanes 2–5 represent treatment times of 5, 10, 30, and 60 min.

To investigate DNA damage in BHS, genomic DNA extracted from bacteria was analyzed using agarose gel electrophoresis. DNA isolated from bacteria treated with chlorine dioxide is degraded, whereas DNA from controls is intact and shows up as a single band in the gel in all experiments (Figure 8). DNA fragmentation also increased with an increasing treatment time of the chlorine dioxide solution. Cumulatively, the results suggest that the chlorine dioxide solution caused DNA degradation in BHS.

## 4. Discussion

Beta-hemolytic *Streptococcus* CMCC 32210 is a standard strain in the Technical Specification for Disinfection and was therefore chosen as the representative for this pilot study. Before studying the bactericidal mechanisms of chlorine dioxide against streptococci, we determined the MBC values of chlorine dioxide against 12 different concentrations of BHS using the checkerboard method. The checkerboard method is an improvement on the micro-broth dilution method and has the advantage of being simple and rapid [30]. The appropriate chlorine dioxide concentrations were selected according to the different requirements of streptococcal concentrations for different tests, and the changes in the indicators of BHS under different chlorine dioxide effects were compared under conditions independent of the streptococcal concentration. 

**Figure 9:**
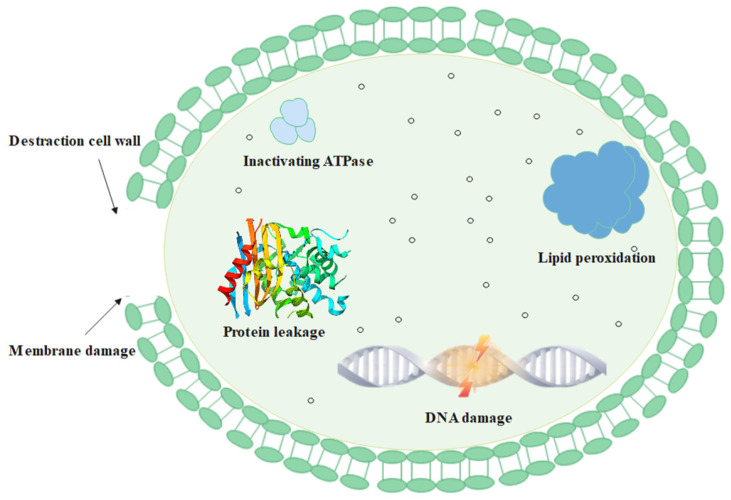
Bactericidal mechanism of chlorine dioxide against BHS.

Our research has revealed that chlorine dioxide acts directly on the cell membrane of BHS, disrupting its structure and increasing permeability. This causes the bacteria to lose their basic structural functions and ultimately results in their death. Additionally, the oxygen radicals present in chlorine dioxide are highly oxidizing and can disrupt the integrity and function of BHS, including proteins, ATPases, lipid peroxidation, and DNA. Thus, there are multiple ways in which chlorine dioxide can affect streptococcal cells, leading to their death (as depicted in Figure 9). 

Disinfectants can produce a bactericidal effect by inhibiting the synthesis of cell wall components, degrading DNA and RNA, denaturing proteins, and interrupting metabolic pathways [35]. Chlorine dioxide readily adsorbs onto the bacterial cell wall, preventing the use of proteins by bacteria [36]. However, studies have shown that the primary lethal damage to *E. coli* O157:H7 by chlorine dioxide is confined to the intracellular area, with no significant damage to the cell wall [19], possibly due to low concentration. Structural changes in *P. aeruginosa* were observed under SEM after treatment with Sp-LECin, indicating membrane disruption and leakage of contents, while no significant changes were found in BHS at 10 mg/L chlorine dioxide treatment, but significant damage was observed at 50 mg/L [37]. We speculate that the disruption of the BHS cell wall and membrane may be one of the mechanisms by which chlorine dioxide kills BHS.

Leakage of cell contents is a key indicator of cell membrane breakage [38], and our SEM results suggest that cell wall damage may lead to this leakage. Chlorine dioxide can generate asymmetric chlorine superoxide radicals (ClOO) [39], which have strong oxidizing effects. This strong oxidant can severely oxidize various proteins on the cell membrane, preventing its normal function [40]. Noss et al. [41] found that six amino acids could be rapidly oxidized under isolated conditions, which may relate to microbial inactivation. Inactivation of microbial proteases by chlorine dioxide was demonstrated by Ogata et al. using yeast 6-phosphoglucose dehydrogenase and bovine serum proteins as a model [42]. Our study showed that increasing chlorine dioxide concentration increased the permeability of the BHS cell wall and membrane, resulting in leakage of intracellular proteins to the outside.

A comparison was made of the protein content, MIC, and MBC values of *Escherichia coli* and *Staphylococcus aureus* before and after treatment with GEO, which showed protein leakage and disruption of bacterial cell membranes. [43]. However, our study investigated the effect of different concentrations of chlorine dioxide on the protein content of BHS at varying exposure times, demonstrating that the bacterial outer membrane was disrupted and that significant protein leakage occurred at chlorine dioxide concentrations of 50 mg/L and 100 mg/L. However, there was no significant protein leakage at low concentration conditions. Chlorine dioxide can increase the permeability of the cell membrane, leading to the leakage of small molecules within the cell [44]. Na^+^/K^+^-ATPase, an important ion pump for maintaining ionic and osmotic homeostasis in cells, may be affected by oxidative denaturation and structural changes caused by chlorine dioxide, resulting in increased permeability of the bacterial outer membrane [45]. The oxidative denaturation and structural changes in enzyme proteins or carrier proteins may contribute to the enhanced permeability of the bacterial outer membrane.

Lipids play an indispensable role in maintaining the structural integrity of cells, as they are the main component of cellular membranes [46]. Chlorine dioxide is a strong oxidizing agent that can induce lipid peroxide production by oxidation of polyunsaturated fatty acids in bacterial cell biofilms via oxygen radicals. This suggests that lipid oxidation may also be a mechanism by which chlorine dioxide kills BHS [47]. The results of the MDA content measurement showed that the streptococci treated with chlorine dioxide experienced a lipid oxidation reaction, indicating an increase in MDA content.

The specific UV absorption of deoxyribonucleotides at 260 nm is due to the conjugated double bonds on purine and pyrimidine bases, and disruption of these conjugated double bonds leads to decreased UV absorption [48]. After treatment with chlorine dioxide, there was a significant decrease in peak area at 260 nm, indicating significant degradation of DNA, and the UV absorption of all four deoxyribonucleotides was strongly affected [49]. These results suggest that the concentration of chlorine dioxide may be related to the observed degradation of DNA.

## 5. Conclusions

Cumulatively, we have found that there may be multiple targets in which BHS can be killed. That is, oxygen radicals from chlorine dioxide can cause oxidation of lipids and proteins in streptococcal biofilms, leading to increased permeability of the biofilm and possible death of BHS due to extravasation of the contents. Protein oxidation leads to the inactivation of Na^+^/K^+^-ATPase and Ca^2+^/Mg^2+^-ATPase in the cell membrane, which subsequently degrades bacterial DNA causing the death of BHS (Figure 9). The mechanism underlying the disruption of cellular walls warrants further investigation. Our study suggests that chlorine dioxide disinfectant products have a multi-target and multi-pathway antibacterial mechanism, which could be developed into a novel antimicrobial agent with potential applications in public health and human health.

## Data Availability

The raw data used and/or analyzed during the current study will be available from the corresponding author on reasonable request.
